# Supplementary material for: Structural basis for the disaggregase activity and regulation of Hsp104
Source: eLife. 2016 Nov 30;5:e21516. doi: 10.7554/eLife.21516 (PMC5130295; doi:10.7554/eLife.21516)
Supplement: Supplementary file 1. — DOI: http://dx.doi.org/10.7554/eLife.21516.027 [file elife-21516-supp1.docx]

**Supplementary file 1: Published Hsp104 and ClpB mutants with repressive or hyperactive phenotype**

|  | **Mutation** | **Comments** | **Ref.** |  |
| --- | --- | --- | --- | --- |
| **Hyperactive mutants** | | | | |
| K374 | ClpB, R356C  Hsp104, K358E | salt-bridge with E498, stabilizes the repressed state by tethering CCD and AAA1S | ([Oguchi et al., 2012](#_ENREF_38), [Lipinska et al., 2013](#_ENREF_33)) | |
| E376 | ClpB, E358C | salt bridge with K494, stabilizes the repressed state by tethering CCD and AAA1S | ([Oguchi et al., 2012](#_ENREF_38)) | |
| I442 | Hsp104, V426L | vdw contact to A517*, linking CCD-CCD* | ([Jackrel et al., 2014](#_ENREF_25)) | |
| E443 | ClpB, E425A | polar interaction with Q520* linking CCD-CCD* | ([Oguchi et al., 2012](#_ENREF_38)) | |
| E481-mod | Hsp104, N467-x | insertion of a Strep-tactin binding motif or T4 lysozyme disrupts CCD-AAA1L/AAA1S contacts | ([Lee et al., 2013](#_ENREF_29), [Lee et al., 2010](#_ENREF_30)) | |
| R484 | ClpB, K466C | salt bridge with D247 stabilizing CCD-AAA1L contact | ([Oguchi et al., 2012](#_ENREF_38)) | |
| K494 | ClpB, K476C | salt-bridge with E376, stabilizes the repressed state by tethering CCD and AAA1S | ([Oguchi et al., 2012](#_ENREF_38)) | |
| E498 | Hsp104, D484K | salt bridge with K374, stabilizes the repressed state by tethering CCD and AAA1S | ([Lipinska et al., 2013](#_ENREF_33)) | |
| R509 | Hsp104, R495M | main-chain interactions with helix 4 of AAA1L | ([Wendler et al., 2007](#_ENREF_64), [Biter et al., 2012a](#_ENREF_3)) | |
| D512 | Hsp104, D498V | salt bridge with R515 stabilizing CCD structure | ([Jackrel et al., 2014](#_ENREF_25)) | |
| H513 | ClpB, L495D | vdw contacts with I442* contributing to CCD-CCD* interface | ([Haslberger et al., 2007](#_ENREF_19)) | |
| A517 | ClpB, S499D  Hsp104, A503X | close vdw contact with I442*, A446* and H445*, stabilizes the repressed state by tethering CCD and CCD* | ([Schirmer et al., 2004](#_ENREF_50), [Haslberger et al., 2007](#_ENREF_19), [Jackrel et al., 2014](#_ENREF_25), [Lee et al., 2005](#_ENREF_32)) | |
| D518 | Hsp104, D504C/V | salt bridge with H445*, tethering CCD and CCD* | ([Jackrel et al., 2014](#_ENREF_25)) | |
| Y521 | ClpB, Y503X  Hsp104, Y507X | vdw contacts with adjacent CCD*, this tyrosine is also implicated in Hsp70 binding | ([Oguchi et al., 2012](#_ENREF_38), [Haslberger et al., 2007](#_ENREF_19), [Jackrel et al., 2014](#_ENREF_25), [Desantis et al., 2014](#_ENREF_10)) | |
| D559 | Hsp104, N539K | polar contacts with K538 and R423 stabilizing CCD/AAA1S interface | ([Jackrel et al., 2014](#_ENREF_25)) | |
| **Repressed mutants** | | | |  |
| R435 | Hsp104, R419M | isolated residue at CCD/AAA1L* interface | ([Wendler et al., 2007](#_ENREF_64), [Biter et al., 2012a](#_ENREF_4))] | |
| E450 | ClpB, E432A | located at the tip of motif-1 of the CCD | ([Oguchi et al., 2012](#_ENREF_38)) | |
| R458 | Hsp104, R444M | located near the tip of motif-1 modulating the E443-Q520 interaction at CCD-CCD* interface | ([Wendler et al., 2007](#_ENREF_64), [Biter et al., 2012a](#_ENREF_4)) | |
| E498 | ClpB, E480C | located at the CCD/AAA1S interface, possibly destabilizing the E373-K374-R501 interplay | ([Oguchi et al., 2012](#_ENREF_38)) | |
| H513 | Hsp104, T499I | contributes to CCD/CCD* interface, insertion of Ile may strengthen hydrophobic interactions with Ile442* and Ala517 | ([Schirmer et al., 2004](#_ENREF_50)) | |
