# Supplementary figures and images for: Structural basis for the disaggregase activity and regulation of Hsp104
Source: eLife. 2016 Nov 30;5:e21516. doi: 10.7554/eLife.21516 (PMC5130295; doi:10.7554/eLife.21516)

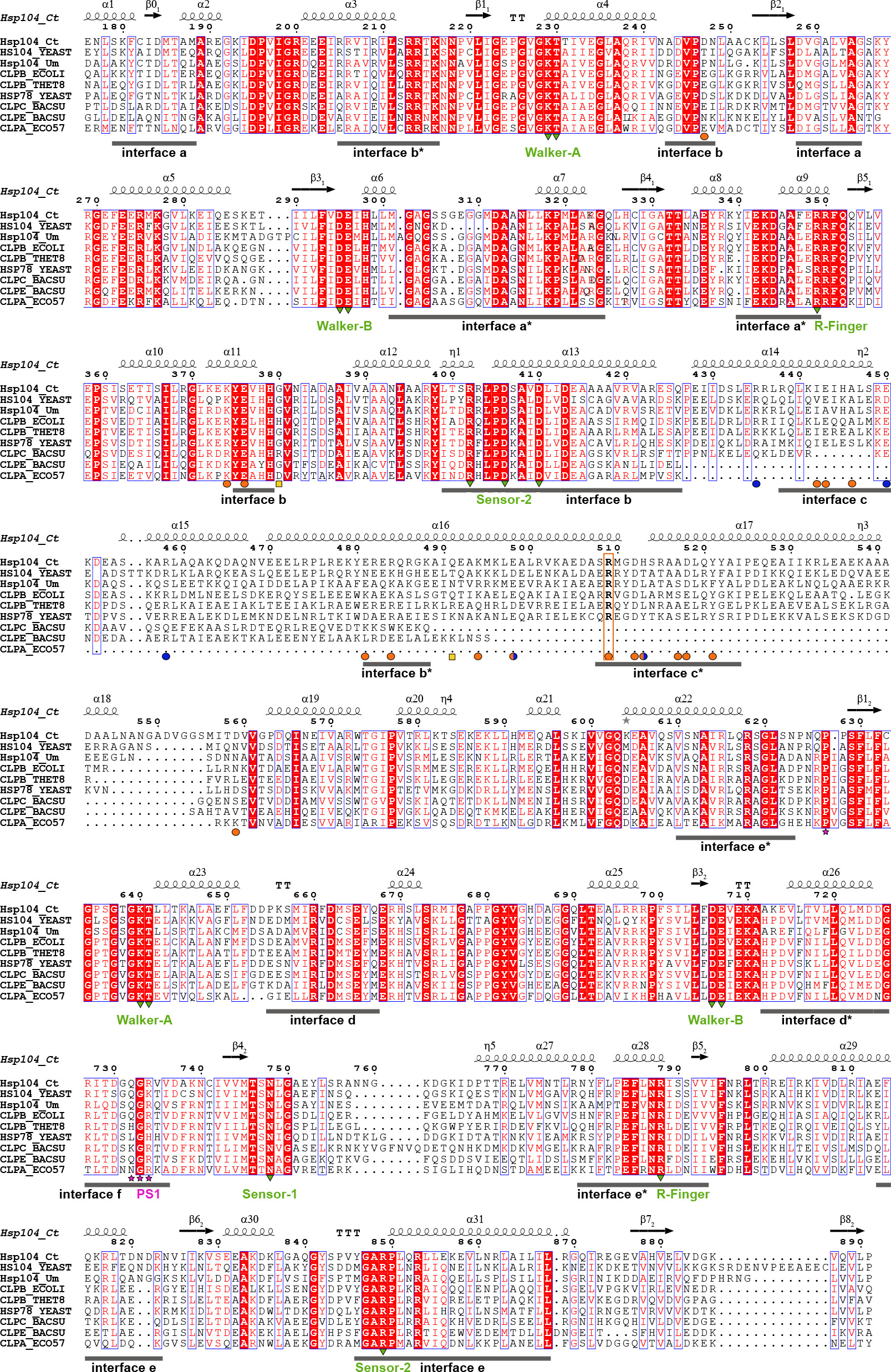

Supplement: Supplementary file 2. — DOI: http://dx.doi.org/10.7554/eLife.21516.028 [file elife-21516-supp2.jpg]
